# Histones: The critical players in innate immunity

**DOI:** 10.3389/fimmu.2022.1030610

**Published:** 2022-11-21

**Authors:** Xia Li, Youyuan Ye, Kailan Peng, Zhuo Zeng, Li Chen, Yanhua Zeng

**Affiliations:** ^1^ Hunan Provincial Key Laboratory for Special Pathogens Prevention and Control, Institute of Pathogenic Biology, Hengyang Medical College, University of South China, Hengyang, Hunan, China; ^2^ Department of Dermatology and Venereology, The First Affiliated Hospital, University of South China, Hengyang, Hunan, China

**Keywords:** histones, innate immunity, DAMPs, AMPs, histone modification

## Abstract

The highly conserved histones in different species seem to represent a very ancient and universal innate host defense system against microorganisms in the biological world. Histones are the essential part of nuclear matter and act as a control switch for DNA transcription. However, histones are also found in the cytoplasm, cell membranes, and extracellular fluid, where they function as host defenses and promote inflammatory responses. In some cases, extracellular histones can act as damage-associated molecular patterns (DAMPs) and bind to pattern recognition receptors (PRRs), thereby triggering innate immune responses and causing initial organ damage. Histones and their fragments serve as antimicrobial peptides (AMPs) to directly eliminate bacteria, viruses, fungi, and parasites *in vitro* and *in vivo*. Histones are also involved in phagocytes-related innate immune response as components of neutrophil extracellular traps (NETs), neutrophil activators, and plasminogen receptors. In addition, as a considerable part of epigenetic regulation, histone modifications play a vital role in regulating the innate immune response and expression of corresponding defense genes. Here, we review the regulatory role of histones in innate immune response, which provides a new strategy for the development of antibiotics and the use of histones as therapeutic targets for inflammatory diseases, sepsis, autoimmune diseases, and COVID-19.

## Introduction

The innate immune system, composed of immune organs, immune cells and immune molecules, is the first line of defense against pathogens and host tissue damage. The innate immune cells such as dendritic cells (DCs), macrophages, natural killer cells, neutrophils, and so on are important parts of the human immune system, which participate in the first non-specific immune responses against invading pathogens (1). Innate immune cells recognize a variety of pathogen-associated molecular patterns (PAMPs) or damage-associated molecular patterns (DAMPs) through pattern recognition receptors (PRRs) expressed on the cell surface, and the activated PRRs transmit signals to the corresponding intracellular system through intracellular signal transduction pathways, resulting in an immune response to external stimuli. Activated PRRs play a vital role in resisting pathogen invasion and maintaining balance in the immune system (2, 3).

As early as 1884, Kossel first discovered histones. With the in-depth study of histones, the roles of histones in biology have attracted more and more attention from researchers. It is well known that histones are the main components of nucleosomes, and they participate in the packaging and arranging of DNA into functional units (4). However, there is increasing evidence that histones are also located outside the nucleus, such as in cytoplasm, and cell surface, as well as in the extracellular fluid, and that these “extranuclear” histones can enhance host defense functions and may contribute to inflammatory responses (5). For example, in the cytoplasm, histones bind to lipid droplets to fight intracellular bacteria (6). In addition, histones also act as cytosolic sensors of viral dsDNA to inhibit viral proliferation (7). One of the most obvious *in vivo* environments in which histones exert their antimicrobial effects *in vivo* is as part of NETs (8). In addition to their antibacterial role *in vivo*, some studies showed that histones and their fragments can act as antimicrobial peptides (AMPs) — critical components of the ancient innate immune system that directly eliminate bacteria, viruses, fungi, and parasites *in vitro*. Histones on cell membranes usually function as protein receptors. Our previous study confirmed that histone H2B located on the cell membrane of human urinary epithelial cells is the receptor of the adhesion protein MgPa of Mycoplasma genitalium (Mg). The combination of MgPa and H2B can mediate the adhesion of Mg and even invade urethral epithelial cells (9). Histones as plasminogen receptors on the surface of the phagocytosis membrane bind to plasminogen to affect the migration and aggregation of inflammatory cells (10). Histones can be passively or actively released from cells during cell damage or signaling. At this time, histones act as DAMPs to promote immune cell activation, inflammasome formation, and pro-inflammatory cytokine release, thereby causing cytotoxicity and immune stimulation and aggravating tissue damage (11, 12). High and low circulating histone levels are associated with the severity or adverse outcomes of several pathophysiological processes, such as inflammatory diseases, sepsis, trauma, autoimmune diseases, and COVID-19 (13–15). Studies have shown that anti-histone antibodies and several commercially available drugs (e.g., heparin) effectively neutralize histone cytotoxicity (16, 17). Therefore, extracellular histones can not only be used as biomarkers for the prognosis and severity of human diseases, but also be expected to become molecular targets for disease treatment.

The signaling pathways of innate immune cells sensing pathogens and initiating innate immune responses are strictly regulated at different levels, including epigenetic regulation. Histone modification is a kind of epigenetic regulation, which mainly occurs in the tail of the amino-terminal of histone protruding from the surface of the nucleosome. Recently, a growing number of studies have shown that histone modifications are essential for generating the necessary cell lineage and gene expression in innate immune response (18). Histone modification participates in the expression regulation of many essential innate immune genes, which affects the recognition and response of innate immune cells to pathogens and the occurrence of related diseases (19–21).

Here, we review recent progress in that histones act as DAMPs to mediate injury and trigger innate immunity. In addition, we discuss the innate immunity of histones associated with phagocytes, the antimicrobial activity of histones, and the influence of post-translational modification of histones on innate immune response and expression of corresponding defense genes. This provides a refreshing perspective for the revitalization of the current antibiotic development strategy and further reveals the occurrence and development mechanism of infection, inflammation, and autoimmune diseases.

## Histone and nucleosome structure

Genomic DNA in eukaryotic cell nuclei is highly restricted, folded, and dense in chromatin formed by dynamic aggregation of histone and non-histone proteins (4). Histones are highly conserved basic proteins that regulate and organize DNA. Five types of histones have been identified, among which H2A, H2B, H3, and H4 are referred to as “core histones” and H1 is “linker histones” (22). The positive charge of histone combines with negatively charged DNA to form the basic unit of chromatin referred to as the nucleosome (22). The nucleosome is formed by wrapping a 147bp DNA fragment around a histone octamer, which consists of a central tetramer composed of two histone H3 molecules and two histone H4 molecules, and two dimers consisting of one histone H2A molecule and one histone H2B molecule. The linker histone H1 binds to DNA in the nucleosome to form a complete nucleosome or chromosome and stabilize the higher-order chromatin structure (23). Each core histone has a central “histone folding” domain and two tails densely packed with basic Lysine and Arginine residues. These amino-terminal sites are flexible and affected by many post-translational modifications associated with transcriptional activation, silencing, chromatin assembly, and DNA replication, which is required for gene regulation and gene replication (24). There is increasing evidence that histone modification is a key mechanism for regulating the expression of important innate immune genes, which play an important role in host perceive and response to microbial pathogens (21).

## Histones act as the damage-associated molecular patterns

**Figure 1:**
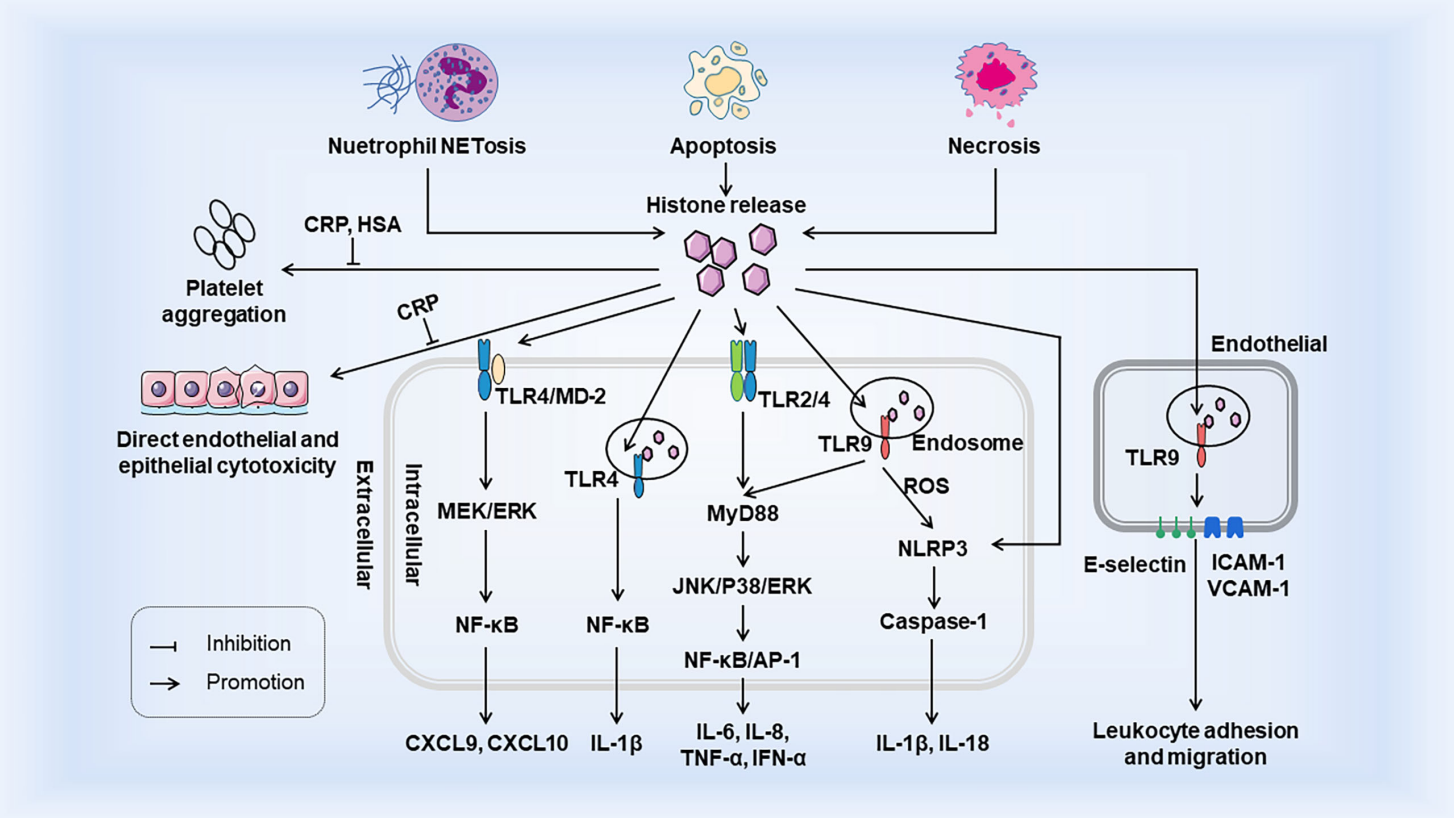
Histones release and its immunostimulatory effects as DAMPs. Histones are released from tissue cells by NETosis, apoptosis, and necrosis. Extracellular histones bind to TLR to trigger intracellular MyD88-dependent signaling pathways and promote the release of cytokines and chemokines, which trigger inflammatory responses and the recruitment and migration of immune cells. Activation of TLR9 leads to the release of ROS, which activates the NLRP3 inflammasome, Caspase1, and further immune cell recruitment. Extracellular histones also induce the surface expression of three endothelial adhesion molecules (E-selectin, ICAM-1 and VCAM-1) through TLR9, thereby facilitating promoting leukemia cell adhesion and migration. Extracellular histones induce endothelial and epithelial cell damage and platelet aggregation, but both are inhibited by CRP. In addition, HSA also inhibits histone - induced platelet aggregation. Both CRP and HSA protect the body from the toxicity of histones released into circulation. HSA, Human Serum Albumin; CRP, C-reactive protein.

Histones are generally confined to the nucleus, but can be released actively or passively in the event of cell injury, stress, or necrosis. When histones are released from nucleosomes, extracellular histones act as DAMPs to interact with TLRs, complements, and cell membrane phospholipids (25, 26), and then exert damage in three ways: (a) as chemokine or inducing chemokine release; (b) inducing adjacent cells and immune cells to release cytokines; and (c) direct cytotoxicity. The occurrence of the innate immune system relies heavily on the interaction of PRRs with PAMPs or DAMPs (2). Extracellular histones belong to DAMPs which also activate innate immunity response similar to PAMPs and promote immune cell maturation. Histones release and its immunostimulatory effects as DAMPs are shown in **Figure 1**.

### TLR-dependent pro-inflammatory cytokine/chemokine release

When histones are released extracellular from necrotic tissues or cells, they act as DAMPs through TLRs, MyD88, NF-κB, and NLRP3 inflammasomes to act on intracellular signal transduction and thus mediate cytokine production and trigger innate immunity (26). Tsourouktsoglou et al. showed that chromatin DNA recruits TLR4 to endosomes containing internalized chromatin after histone binding and activation of TLR4, thereby acting as signaling mediators of inflammation to promote cytokine release in infections and sterile diseases (27). Histones specifically induce monocytes to release chemokines CXCL9 and CXCL10, and chemokine induction is closely related to the TLR4/myeloid differentiation factor 2 (MD-2) complex on monocytes (28). In addition, *in vivo* experiments in mice showed that the elevation of extracellular histone not only increased the level of CXCL10, but also led to leukocyte recruitment in a TLR4-dependent manner (29). Extracellular arginine-rich histones, namely H3 and H4, also activate vascular endothelial cells and induce expression of endothelial adhesion molecules such as E-selectin, ICAM-1, and VCAM-1, thus increasing leukocyte adhesion, rolling, and migration in a TLR9 dependent manner (30–32). Functionally, when renal tubular epithelial cells die, histones are released outside the cells and interact directly with TLR2 and TLR4 to induce MyD88, NF-κB, and MAPK signal transduction. In addition, direct injection of histone into the renal artery of rats can lead to necroinflammation and the release of related inflammatory molecules, such as IL-6, TNF-α, and inducible nitric oxide synthase (16). These effects were attenuated in TLR2 and TLR4 knockout mice. Hiroki Kawano et al. showed that histones induce ARPE-19 cells to produce IL-8 and IL-6 through TLR4 and ERK1/2 and p38 MAPK-dependent pathways, and inhibit ERK1/2 and p38MAPK to reduce cytokine release (33). In addition to its effect on TLR, histones also activate bone-marrow-derived DCs through NLRP3 inflammasome, a critical system inducing IL-1β and IL-18 production in the innate immune system. Allam et al. demonstrated that histones induce IL-1β secretion and the production of sterile inflammation in an NLRP3-Asc-Caspase-dependent manner, and the deletion of mouse NLRP3 gene significantly reduces both histone-induced IL-1β secretion and neutrophil recruitment (34). Another research also confirmed histones activate the NLRP3 inflammasome in Kupffer cells and thus induce secretion of the downstream molecules such as caspase-1, pro-lL-1β, and pro-IL-18 through TLR9-dependent ROS production during liver injury, thereby promoting the innate immune response after I/R injury (35). However, histones and NETs directly initiate IL-1β release from macrophages before inflammasome activation, thereby driving sterile inflammation (36). This suggests that histones can act directly as a priming signal or as DAMP to activate the inflammasome to stimulate IL-1β secretion. In summary, in these examples of “sterile inflammation”, histones play a crucial role in initial organ damage and innate immune activation. At the same time, it also reveals a novel mechanism of organ damage in which neutralizing histones may benefit sterile inflammation-induced pathology.

### Extracellular histones related to clinical conditions

In the case of infection, extracellular histones can be used as bactericidal proteins to locally limit the spread of infection and initiate tissue regeneration and repair. On the other hand, circulating extracellular histones can bind to double stranded (ds) DNA or high motility group box 1 (HMGB1) in cells to form DAMPs, which initiate and persist systemic cytotoxic damage through a non-infectious inflammatory response (27, 37). Xu et al. first proved that the cytotoxicity of extracellular histones is non-specific and can damage host cells in sepsis experiments (38). Extracellular histone H4 can also cause the dissolution of the smooth muscle cell (SMC) membrane, which triggers arterial tissue injury and chronic inflammation (39). Extracellular histone is the primary mediator of organ dysfunction and death in patients with sepsis, which can cause microcirculatory dysfunction. In septicemia or major trauma, histone release mainly affects endothelial cells and even leads to fatal organ dysfunction (40). This may be caused by the interaction of histone with the negatively charged phosphate group of the phospholipid bilayer and the influx of abnormal ions. Notably, c-reactive protein can competitively bind histones with phospholipid liposomes, prevent histones from entering the cell membrane and prevent calcium influx, thus reducing histone-mediated toxicity (41, 42). In addition, in an animal model of sepsis, heparin has been shown to bind to histone and neutralize its cytotoxicity (43). Other studies have shown that myeloperoxidase derived from neutrophils can inhibit the release of histones, thus reducing septicemia (44). In sterile and infectious acute inflammatory disease models, continuous histone administration can aggravate end-organ damage, such as kidneys (16), liver (35), heart (45), and lungs (46). Similarly, histone targeting or histone-specific therapy can partially ameliorate organ damage.

Several studies showed that the severity of COVID-19 is associated with circulating extracellular histone levels (15, 47–49). Circulating histone is the mediator of epithelial/endothelial cell injury, which may trigger and spread inflammatory storms and thrombosis, increase the severity of the disease and reduce the survival rate of patients (50–52). Compared with healthy controls, the level of free histones (related to DNA fragments) in the plasma of patients with severe COVID-19 was significantly higher (47, 49). Huckriede et al. further demonstrated the harmful role of extracellular histone in the progression of COVID-19 disease (47). It is worth noting that the presence of citrulline histone H3 positive neutrophils in pulmonary micro thrombosis suggests that citrulline histone H3 may also play a role in COVID-associated thrombotic inflammation (42, 53, 54). Therefore, monitoring circulating histone concentrations, including unmodified and citrulline histone H3, may be a useful tool for early prediction of a higher risk of progression of adverse disease during admission and hospitalization in SARS-CoV-2-infected patients.

## Histones and phagocytes-related innate immune response

### Histones and neutrophil extracellular traps

Phagocytes play a crucial role as the building blocks of innate immune defense against infectious pathogens. There is growing evidence suggesting intriguing associations between histones and phagocytes. In addition to binding as DAMPs to PRRs on phagocytes, histones also function as bactericidal components in NETs. NETs, fibrous structures, are decondensed chromatin networks released from activated neutrophils during infection and aseptic inflammation and can capture and eliminate pathogens (55, 56). NETs neutralize, capture and kill bacteria, fungi, viruses, and parasites, but some pathogenic bacteria can avoid Net-induced killing (57). All core histones are present in NETs, accounting for 70% of all NET-related proteins (8). Some research data reported that the linker histone H1 also exists in NETs (58), but other data indicated that H1 could be degraded during NETs formation (59). In unstimulated neutrophils, the content of all core histones is equal. However, in NETs, the concentrations of H2A and H2B are higher than those of H3 and H4 (8). Histones can either act as bactericidal components in NETs or activate neutrophils in a dose-dependent manner to form NETs. The function of histones in NETs is initially unclear, as histones may be merely residual features of neutrophils (60). Nevertheless, the coexistence of histones in NETs scaffolders with human AMPs suggested that histones may act as antimicrobial agents in NETs (61, 62). Importantly, when NETs react with antibodies against histone H2A and H2B, NETs lose almost all potency against Gram-positive and negative bacteria, indicating the importance of histone (58). However, in addition to enhancing host defense, NETs also cause damage to surrounding tissues by increasing pro-inflammatory responses or by themselves. For example, NETs are associated with the pathology of autoimmune diseases (psoriasis, systemic lupus erythematosus, and rheumatoid arthritis), sepsis, chronic obstructive pulmonary disease, and cancer (60). Therefore, there is a need to balance between the inflammatory damage caused by NETs and the benefits of host defense.

### Histones and the activation of neutrophils

Extracellular histone H4 is an activator of neutrophils, which continuously increases the concentration of intracellular calcium by affecting the membrane permeability of neutrophils, leading to respiratory burst response, adhesion, myeloperoxidase (MPO), and IL-8 release (63, 64). Respiratory burst and degranulation responses depend primarily on the elevation of intracellular calcium from extracellular sources. Conversely, the influx of calcium results from histone H4 permeating the neutrophil membrane. Another Arginine rich histone, histone H3, has a similar effect on neutrophil activation, while histone H2A and H2B cannot activate neutrophils (64). These suggest that activating neutrophils is a characteristic of Arginine-rich histones.

### Histones act as plasminogen receptors

Related studies manifested that H2B is a controllable plasminogen receptor on the membrane surface of monocytes, macrophages, and neutrophils. The recognition and binding site of plasminogen is the C-terminal Lysine residue of H2B (10). After binding to H2B, plasminogen is transformed into plasmin, which mediates pericellular proteolysis and thus promotes cell migration and matrix remodeling, which influence the migration and recruitment of inflammatory cells during inflammatory responses (65). It is worth noting that the function of H2B as a plasminogen receptor may be inhibited by histone H2B autoantibodies. For example, autoantibodies against histone H2B on CD4^+^ T lymphocytes are associated with disease activity in HIV-infected individuals. The reduced ability of these cells to use plasminogen to facilitate inflammatory cell migration may increase the susceptibility of HIV individuals to infectious diseases (66). According to reports, more than 70% of patients with autoimmune diseases such as systemic lupus erythematosus have autoantibodies against histone H2B (67), which also explains the increased risk of thromboembolism in patients with autoimmune diseases.

## Antimicrobial activity of histones

The antibacterial role of histones in many different species is increasingly being recognized, including invertebrates (68), chickens (69, 70), frogs (71), fish (72), and mammals such as mice (73), cattle (74) and humans (75). The antibacterial activity of histones was reported as early as 1942 (76). Subsequently, various studies have shown that histones and histone-derived fragments from arthropods to mammals have broad-spectrum antibacterial activity (73, 77). AMPs, also known as host defense peptides (HDPs), are ubiquitous in all living organisms. They can eliminate or destroy pathogenic microorganisms, including bacteria, fungi, viruses, and parasites, and are key components of the host’s innate immune system. There are many biochemical similarities between histones and AMPs. They are cationic proteins with the ability to form α helices and contain a large amount of hydrophobic amino acids, suggesting histones may also have antibacterial properties (78). Histones and their fragments play a vital role in host defense by binding to bacterial nucleic acid, binding to bacterial lipopolysaccharide (LPS, a bacterial membrane component), changing the permeability of bacterial cell membrane, and inhibiting viral binding. **Table 1** summarizes the antibacterial properties of histones and related fragments obtained from various animals.

**Table 1:** Antimicrobial activity of his tones and his tone fragments.

Histone		Origin	Antimicrobial Spectrum					References
			Gram-positive	Gram-negative	Virus	Fungi	Parasites
H1	Full-length	murine	*L.Monocytogenes*, *S.aureus, M.fortuitum*	*E.Coli*, *S.typhimurium*	Unknown	*C. neoformans*	Unknown	(34)
		human	*E. faecalis, S.aureus*, *S. Epidermidis, MRSA*	*P. aeruginosa*, *E. coli, S. typhimurium*	*HPV*,*HIV-1*, *Norwalkvirus*	*C. tropicalis*	Unknown	(7, 35–42)
		fish	*S. aureus*	*E. coli*	Unknown	Unknown	Unknown	(43)
		chicken	*B. subtilis*	*E. coli*	Unknown	Unknown	Unknown	(44)
		wheat	Unknown	Unknown	Unknown	*Aspergillus*, *Fusarium*, *Penicillium*,*Greeneria*	Unknown	(45)
	N-terminal	fish	*B.subtilis*, *L.ivanovii*	*V. alginolyticus*, *E. coli* *A. hydrophila*,*S. enterica*	Unknown	Unknown	Unknown	(46)
	Onchorhyncin II	fish	*L.anguillarum*, *M.luteus*,*P.citreus*	*E. coli*	Unknown	Unknown	Unknown	(47)
H2A	Full-length	human	Unknown	*E. coli*	*HIV-1*, *Norwalkvirus*, *Influenzavirus*	Unknown	*L. amazonensis*, *L. amazonensis*, *L.mexicana*,*L.major*	(40–42, 48, 49)
		calf	*S.aureus*	*S. flexneri*, *S.typhimurium*	Unknown	Unknown	Unknown	(50)
		chicken	*B. subtilis*	*E. coli*	Unknown	Unknown	Unknown	(51)
		fish	*A. Viridans*, *M. luteus* *R. salmoninarum* *P. citreus*,*S. aureus* *B. subtillis*	*A. hydrophila*, *A.salmonicida*, *L. anguillarum*, *E.coli*,*Y.ruckeri*	Unknown	Unknown	Unknown	(52)
		shrimp	*M. luteus*	Unknown	Unknown	Unknown	Unknown	(53)
	Buforin I and Buforin II	frog	*B. subtilis*, *S. aureus*,*S. mutans*	*S. typhimurium*, *E.coli*, *P. putida*	Unknown	*C. neoformans*, *S. cerevisae*, *C. albicans*	Unknown	(54, 55)
	Parasin I	catfish	*B. subtilis*, *S. aureus*, *S. mutans*	*S. Enteritidis* *E. coli*, *P. putida*	Unknown	*C. neoformans*, *S. cerevisae*, *C. albicans*	Unknown	(56)
	Hipposin	fish	*B. subtilis*	*E. coli*	Unknown	Unknown	Unknown	(57, 58)
H2B	Full-length	human	Unknown	*E. coli*	*HPV*, *Norwalkvirus*, *Influenzavirus*	Unknown	*L. braziliensis*, *L. amazonensis*, *L. mexicana*, *L. major*	(39, 41–43, 49)
		murine	*L. Monoctogenes*, *S. aureus*,*M. forutitum*	*E. coli*, *S.typhimurium*	Unknown	*C. neoformans*	Unknown	(34)
		chicken	*B. subtilis*	*E. coli*	Unknown	Unknown	Unknown	(51)
		fish	*B. megaterium*	*E. coli*	Unknown	*C. albicans*	Unknown	(59)
	C-terminal	chicken	*B. subtilis*	*E. coli*	Unknown	Unknown	Unknown	(51)
	N-terminal	human	*M.luteus*, *B.megaterium*	Unknown	Unknown	Unknown	Unknown	(60)
H3	Full-length	human	*S. aureu*	*E. coli*	*Norwalkvirus*, *Influenzavirus*	Unknown	Unknown	(41, 42, 61)
H4	Full-length	human	*S. aureus*, *P. acnes*	Unknown	*Norwalk virus*, *Influenza virus*	Unknown	Unknown	(41, 42, 62)
		calf	*S.aureus*	*E.coli*	Unknown	Unknown	Unknown	(61)
		wheat	Unknown	Unknown	Unknown	*Aspergillus*, *Fusarium*, *Penicillium*,*Greeneria*,	Unknown	(45)
	Histogrannin	calf	*B. subtilis*, *S. aureus*	*E. coli*, *P. aeruginosa*	Unknown	Unknown	Unknown	(63)

### Antibacterial activity

Many studies have shown that core and linker histones have antibacterial activities in various organisms. Linker histone was found in the granules of macrophages and was originally named murine microbial proteins 1 and 2 (MUMP-1, MUMP-2). MUMP exhibits antibacterial activity against various microorganisms, including *S. typhimurium*, *E. coli*, *S. aureus*, *M. fortuitum*, and *L. monocytogenes* (73). The antibacterial activity of histone H1 has been reported in various species from humans to fish. In humans, H1 in the terminal ileum mucosa and the colonic epithelial cells showed antimicrobial activity against *S. typhimurium* and *E. coli*, respectively (75, 79). When histones from epithelial cells reach the lumen, they may represent critical innate antimicrobial defense of the human intestine against luminal bacteria (79). Recombinant human histone H1 showed broad-spectrum antimicrobial activity against Gram-negative and positive bacteria, including drug-resistant strains (80). In fish, histone H1 is an important antibacterial protein in the stomach, intestine, and liver of *Atlantic salmon*, with significant anti-*E. coli* activity. Histone H1, isolated and identified from the testis of *Atlantic salmon*, showed significant antibacterial activity against both Gram-negative and positive bacteria (72). In chickens, H1 in the ovary and oviduct showed antibacterial activity against *B. subtilis* and *E. coli*, indicating the defense of histone H1 against pathogens during ovarian follicle development and ovum formation in fallopian tubes (70).

As an antimicrobial agent, H2A is the most studied member of the histone protein family. H2B was first isolated in 1958 from mouse macrophage line RAW264.7 and proved to have extensive antibacterial properties (73). After purification, low doses of H2A have a strong killing effect on *S. typhimurium*, *S. flexneri*, and *S. aureus* within a short time, and its bactericidal activity is canceled in the presence of anti-histone H2A antibody, which proves that H2A is an effective antibacterial protein (58). H2A and H2B also exist on the surface of the human placental epithelium, protecting the fetus and placenta from microbial infection (89). In addition, H2A and H2B showed antibacterial activity against Gram-positive and negative bacteria in chicken, fish, and shrimp (68, 69, 91). The antibacterial protein H2B isolated from the epidermal mucus of Atlantic cod (*Gadus Morhua*) also showed antibacterial activity against *Candida albicans* (96). Notably, histones H2A and H2B, as well as H3 and H4, bind to the LPS of bacteria, possibly showing antibacterial activity by binding to bacterial membranes under certain conditions. The affinity of histones to LPS was higher than polymyxin B except for H4 (81).

So far, there are relatively few studies on the antibacterial activity of histones rich in Arginine (H3, H4). Although histone H3 has shown antibacterial activity against both *S. aureus* and *E. coli*, little is known about its antibacterial mechanism (98). Lee et al. showed that human cortical cells fully secreted and released H4, which had bactericidal activity against *P. acnes* and *S. aureus*. In addition, histone H4 enhanced the antibacterial effect of free fatty acids in human sebum (99). These results indicate that histone H4 is the main component of the antibacterial action of human sebum cells and plays a vital role in the innate immune defense of the skin. H4 found in calf thymus can also kill *E. coli* and *S. aureus*, which further strengthens the understanding of the antibacterial activity of H4 (98).

### Antifungal activity

Histones also show antifungal properties. Within 10 minutes, only eight micrograms of human epidermal histone H1 killed 90% of *Candida tropicalis* (1x105 CFU). Therefore, when skin Candida albicans infection, histone H1 may form an antifungal barrier in the human epidermis, which prevents Candida from invading deeper than the granular cell layer (100). In addition, the study of antibacterial proteins in mouse macrophages found that H1 and H2B have bactericidal activities not only against a variety of bacteria, but also *Cryptococcus neoformans A* (73). It has also been found that wheat histones H1 and H4 have apparent inhibitory effects on *Fusarium* and some wheat pathogens such as *Aspergillus*, *Penicillium*, and *Greeneria in vitro*, indicating that extracellular histones may enhance the resistance of host plants to these funguses (86). It is worth noting that we still know little about the antifungal mechanism and antifungal spectrum of histones, which needs to be further studied.

### Antiviral and antiparasitic activity

So far, most studies on histone antipathogens have focused on their antibacterial and antifungal activities. However, histones have also been shown to have antiviral and antiparasitic activities. The binding of histones to DNA not only helps regulate the transcriptional activity of genes, but also indirectly protects against viral replication in host cells. In human cells infected with human papillomavirus type 11 (HPV-11), H1 blocked viral replication by binding to the viral genome (82). Another study showed that H2B binds to viral dsDNA, hence activating the natural antiviral pathway and inhibiting viral proliferation (7). Kozlowski et al. found that extracellular H1 and H2A, but not H2B, H3, or H4, significantly inhibited HIV-1 infection at the viral transcriptional level (83). Of course, histones also exhibit antiviral activity through direct action. Both linker histone H1 and core histone showed anti-Norwalk activity by inhibiting *Norwalk virus* attachment to host cells by binding to virus particles and cell membranes (84). For *Influenza viruses*, histones have a strong neutralization effect against H3N2 and H1N1 seasonal strains, but not against pandemic H1N1 strains (85). In summary, all five histones have antiviral activity, but the antiviral activity of H1, H2A, and H2B is weaker than that of H3 and H4. H4 is the most effective histone to inhibit viral infectivity, inhibiting viral uptake and replication by directly interacting with virus particles (85). Notably, histones also have antiparasitic activity. Human histone H2A and H2B kill *Leishmania* promastigotes, and the killing intensity is dose-dependent. Histones also significantly reduced the infectivity of mouse macrophage promastigotes *in vitro* (90).

### Histone-derived fragments

In addition to intact histones, histone fragments and histone derivatives also showed prominent antibacterial activity. The fragmented histone H1 showed antibacterial activity. For example, the N-terminal of histone H1 fragment found in salmon mucus and blood samples showed antibacterial activity against Gram-positive and negative bacteria (87). Oncorhyncin II, the C-terminal fragment of H1 found in the acidic extract of rainbow trout skin secretion, showed antibacterial activity against Gram-positive and negative bacteria and even fungi (88). H2A fragments exert strong antibacterial activity in natural or synthetic form. Buforin I (BF1) and Buforin II (BF2) are H2A fragments isolated from the stomach of the Asian toad. Compared with BF1, BF2 has higher antibacterial activity and can bind to DNA and RNA like H2A, and inhibit the function of bacteria by binding to bacterial nucleic acid (71, 92). Two kinds of AMP related to histone H2A, Parasin I and Hipposin, were found in fish skin. Parasin I is the N-terminal peptide of H2A, which has antibacterial activity against fish-specific bacterial pathogens and helps fish to resist the invasion of microorganisms after injury (93). Hipposin is an AMP derived from histone H2A isolated from the skin mucus of Atlantic halibut and has antibacterial activity against Gram-positive and negative bacteria (94, 95). For the histone H2B fragment, the C-terminal fragment isolated from chicken liver could inhibit the activity of Gram-positive and negative bacteria (69), and the N-terminal fragment in the human wounds and blister fluid inhibited the growth of *Micrococcus luteus* and *Bacillus megaterium* (97). No natural fragment of histone H3 has been found. However, Histogrannin, an H4 fragment isolated from bovine adrenal medulla, can inhibit the activity of ATP-dependent DNA gyrase and thus exhibit antibacterial activity against Gram-positive and negative bacteria (74).

## Histone modification and innate immunity

“Epigenetic” refers to the change of gene activity but independent of DNA sequence. There are four main mechanisms of epigenetic regulation: DNA methylation, chromatin structure regulation, histone post-translational modification, and non-coding RNA regulation (101). Histones post-translational modification typically occurs on amino acid residues, including methylation and acetylation of Lysine and Arginine, phosphorylation of Serine and Threonine, ubiquitination of Lysine, total methylation, citrullination, and ribosylation. These modifications are associated with transcriptional activation, silencing, chromatin assembly, and DNA replication, which can activate or inactivate gene expression and thus regulate cellular behavior. Tissue modification enzymes involved in epigenetic regulation include histone methyltransferases (HMTs), histone demethylases (HDMs), histone acetyltransferases (HATs), and histone deacetylases (HDACs), as well as other proteins (18). Histone modification and regulatory enzymes critically regulate the expression of corresponding defense genes and the occurrence of innate immune responses at multiple levels. Among these histone modifications, acetylation and methylation are the most important in regulating the transcription of pro-inflammatory cytokines.

### Histone acetylation/deacetylation

Histone acetylation is an essential epigenetic mechanism that controls DNA accessibility, chromatin structure, and gene expression. It is dynamically and reversibly regulated by two groups of relative enzymes, namely HDACs and HATs. HATs are involved in specific gene activation in the process of inflammation (21). For example, histone acetyltransferase p300 mediated the acetylation of H3 at Lysine 18 and 27 (H3K18/27ac) to activate the expression of pro-inflammatory genes (102). Nevertheless, H3k9ac and H3K14ac are necessary for transcription factors and Polymerase II (Pol-II) to enter the promoter region. H3k9ac and H3K14ac are downregulated in the promoter region of inflammatory mediator genes, such as CIITA, TNF-α, IL-6, NOS2, and H2EB, under the stimulation of soluble/secretory factor (HSF) of parasite *M. Corti* (21). During macrophage infection, inhibition of HAT activity reduces the secretion of matrix metalloproteinase-1 (MMP-1) and matrix metalloproteinase-3 (MMP-3), which are closely related to the immune pathogenesis of *Mycobacterium tuberculosis* (103).

The role of HDACs and their inhibitors in innate immune pathways has also been gradually discovered. Some studies have linked the development of myeloid cells to specific HDACs (104). For example, HDAC3 interacts with the transcription factor PU.1 and blocks the expression of target genes, thereby negatively regulating the differentiation of myeloid cells (105). However, the expression of HDAC5 was upregulated during the differentiation of human monocytes into macrophages. HDACs also regulate the function of mature macrophages and DCs by controlling the production of inflammatory mediators such as chemokines, cytokines, and matrix metalloproteinases (MMPs). In these cells, the TLR and IFN signaling pathways are especially regulated by HDACs (106). HDACs can act as either positive regulators or negative regulators of TLR signaling. HDACs promote the expression of TLR target genes such as chemokines (e.g., CCL2, CCL7, and CXCL10), cytokines (e.g., IL-6, IL-12, TNF, and IFN-β), and other secreted inflammatory mediators (e.g., MMP-9 and endothelin-1), but its promotion mechanism and the specific HDAC involved are still unclear (107). HDACs also promote the TLR response by regulating the function of transcription factors (HIF-1α and IRF transcription factor family) (106). HDAC1 and HDAC8 suppress the IRF function, while HDAC6 promotes the IRF function to respond to virus challenges. However, class I HDACs possibly exert a negative regulatory effect on TLR response by promoting histone deacetylation and transcriptional inhibition. For example, HDAC1 inhibits the activity of many inflammatory gene promoters induced by TLR and/or pathogens, including IFN-β, COX-2 (108), and IL-12p40 (109). NF-κB signal is activated by the TLR signal, which is the center of inflammatory response. Classic HDACs 1, 2, and 3 negative regulation NF-κB. HDAC1 inhibits inflammatory gene promoters by inhibiting NF-κB p65 and mediating NF-κB p50 homodimer (110). HDAC2 blocks the activation of inflammatory cytokine genes in macrophages by interacting with transcriptionally activated metastatic tumor antigen (MTA) 1 (111). HDAC3 deacetylates p65 subunit and promotes the interaction between the p65 and NF-κB inhibitor-α (IκBα) to indirectly and negatively regulate NF-κB signal transduction (112). In addition, the effect of HDAC inhibitors on the innate immune system has also received widespread attention. Some studies showed that HDAC inhibitors can down-regulate the expression of multiple host defense genes, including PRR, chemokines, growth factors, and transcriptional regulators, and inhibit the expression of critical antibacterial cytokines and co-signal molecules in macrophages and DCs (107). *In vivo*, HDAC inhibitors can increase sensitivity to bacterial and fungal infections, but have protective effects on toxic and septic shock (113). These indicated HDAC inhibitors play multiple regulatory roles in different functional states of the organism. These studies confirmed the significant role of histone acetylation in regulating innate immune gene expression.

### Histone methylation/demethylation

**Figure 2:**
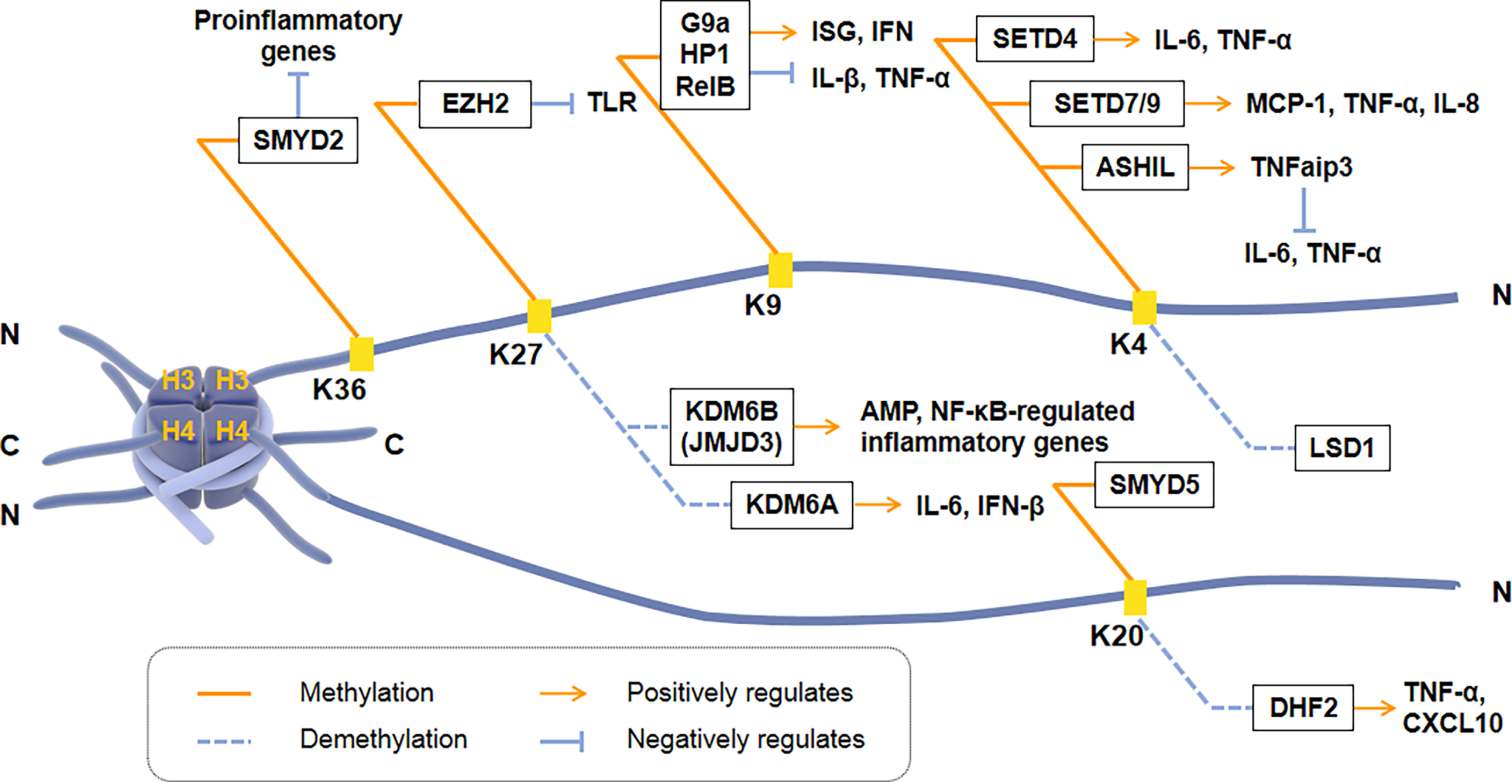
Regulation of transcriptional activation of inflammatory cytokines by histone methyltransferases and demethylases. Histone methylation occurs at the N-terminus of Lysine or Arginine residues of Histone H3 and H4, including H3K4, H3K9, H3K27, H3K36, and H4K20. SMYD2, EZH2, G9a, SETD4, SET7/9, ASH1L and SMYD5 are histone methyltransferases. KDM6B (JMJD3), KDM6A, LSD1 and DHF2 are histone demethylases. ISG, IFN-stimulated gene; TNFaip3, a negative regulator of TLR signaling pathway.

Histone methylation occurs at the N-terminus of lysine or arginine residues of histone H3 and H4, including H3K4, H3K9, H3K27, H3K36, and H4K20. Histone methylation is closely regulated by HMTs and HDMs and plays a vital role in the transcription of inflammatory cytokines [**Figure 2**]. H3K4me3 can regulate the induction of pro-inflammatory genes in macrophages. For example, the methyltransferase SETD4 directly activates H3K4 methylation to positively regulate the expression of IL-6 and TNF-α in macrophages (114). Another H3K4 methyltransferase, SET7/9, also has a positive regulatory effect on TNF-α induced MCP-1, and IL-8 gene subsets in THP-1 macrophages. However, histone H3K4 methyltransferase ASH1L inhibited NF-κB signaling downstream of the TLR by promoting the expression of TNFaip3 (a negative regulator of TLR signaling pathway), thereby inhibiting macrophage production of IL-6 and TNF-α (115).

H3K9 methylation is correlated with promoters of inducible inflammatory cytokines. Histone methyltransferase G9a promotes the recruitment of heterochromatin protein 1 (HP-1) and inhibits TNF-α expression by catalyzing H3K9 di-methylation of TNF-α promoter in THP-1 macrophages (116). Another research showed that G9a interacts with the NF-κB transcription factor RelB to induce endotoxin tolerance in macrophages. HP1 and G9a form a complex at the IL-1β promoter, while RelB decomposes the complex and removes transcriptional silencing, which ultimately affects the production of IL-1β in innate immune cells (116). Fang et al. found that H3K9me2 establishes cell-specific inducibility of IFN and IFN-stimulated gene (ISG). In addition, the knockout of the H3K9 methyltransferase G9a gene increased the ability of fibroblasts to produce interferon and its resistance to viruses (117, 118). These showed that H3K9me2 and G9a are the critical regulators of innate antiviral immunity.

H3K27 methylation also regulates the transcriptional response of innate immune cells. KDM6B (JMJD3) is the first reported Lysine demethylase that controls inflammation, responsible for the demethylation of di-methylated and tri-methylated H3K27 (119). The down-regulation of JMJD3 in THP1 monocytes deregulates the transcriptional regulation of the NF-κB target genes network, including cytokines, chemokines, and immune receptors (120, 121). Another Lysine demethylase, KDM6A, specifically promotes the transcription of INF-β through increasing enhancers activity during virus infecting macrophages and of IL-6 through demethylating H3K27me3 (122). During ontogeny, H3K27 methylation and JMJD3 are closely related to the expression of AMP in skin. Knockdown of JMJD3 in the skin keratinocytes (KC) elevates H3K27me3 levels, while the expression of AMPs HBD-3, S100A7, S100A8, S100A9, and actinomycin downregulated significantly (123).

H4K20me3 is the crucial point to inhibit the expression of TLR4 target genes in macrophages, indicating that inflammation can be regulated by co-regulators activating or inhibiting specific histones. H4K20me3 methylation/demethylation is required for signal-dependent regulation of the subset inflammatory response genes, catalyzed by SMYD5 and PHF2, respectively (21). SMYD5 maintains H4K20me3 on TLR4 response promoters (IL-1, TNF-α, CCl4, and CXCL10) by binding to NCoR corepressor complexes. Two hours after TLR4-mediated activation, down-regulation of PHF2 resulted in an increase in the inflammatory promoter H4K20me3, and a decrease in the activation of inflammatory genes such as TNF-α and CXCL10 (124). In addition, SMYD2 specifically facilitates H3K36 di-methylation on the IL-6 supporters, thus suppressing the production of pro-inflammatory cytokines induced by macrophage activation (125).

### Histone citrullination

Histone citrullination is a post-transcriptional modification catalyzed by the peptide arginine deiminase (PAD) family, including five isozymes (PAD1-4 and PAD6) with different tissue-specific targets (126). PAD4-dependent citrullination of histone H3 and H4 in neutrophil nuclei is a key process that allows chromatin decryption and NETs excretion, constituting a unique form of cell death called NETosis (57). Although there is no specific marker for NETs, citrullinated histone 3 (H3Cit) is considered to be the key component to determine the existence of NETs and is deemed to be a biomarker of inflammation (127). H3Cit also increases in various inflammatory states in mice and humans. H3Cit can directly participate in inflammatory injury by destroying the microvascular endothelial barrier (128). H3Cit may have a pathogenic effect on tissue damage because it has been shown that injection of H3Cit antibodies or PAD inhibitors can improve the prognosis of systemic inflammation (129). The activation of inflammatory bodies in neutrophils and monocytes/macrophages in COVID-19 patients also showed that the formation of inflammatory bodies was significantly related to the citrullination of H3 in the nucleus (54). In addition, histone H4 can also induce NET formation in a calcium-and PAD4-dependent manner. However, citrullinated histone H4 induced less calcium influx, resulting in a decrease in NET formation (130). This suggests that citrullinated histone H4 may act as a brake in the pathological process of NETs and slow down the vicious circle between histone H4 and NETs. Since histone H3 citrullination is necessary for NETs formation, histone citrullination is also inextricably linked with thrombosis, atherosclerosis, autoimmunity, tissue repair in diabetes, and cancer through NETs (27, 131–133). These data show that the circulating level of H3Cit in the blood can be used as a marker of inflammation, which provides a reliable standard for the diagnosis and prognosis of various inflammatory diseases.

### Other modifications of histones

The E3 ubiquitin ligase RNF20 drives monoubiquitylation of histone H2B, which contributes to target inhibitory p50 homodimers and increases H3K9me3 on the pro-inflammatory cytokine subset promoter to inhibit colon inflammation (134). Histone H2A monoubiquitylation catalyzed by DZIP3 inhibits the transcription of chemokines, crucially regulating the migration response of macrophages to TLR activation (135).

## Conclusions and outlook

### Histones act as therapeutic targets for disease

Histones are essential components of nuclear material that help to form chromatin and act as a control switch for DNA transcription. In addition to regulating transcription, the roles of extracellular histones in the pathology of serious diseases such as stroke, sepsis, and systemic lupus erythematosus have recently attracted considerable attention. High concentrations of histones can cause toxic reactions and systemic inflammatory reactions, which even lead to life-threatening complications (41). However, lower extracellular histones act as DAMPs to participate in the immune defense or wound healing by triggering local host responses. In aseptic inflammation, histones as DAMPs not only play an essential role in innate immune activation but also cause initial damage to the corresponding organs. These findings indicated the dual role of extracellular histones in both systemic and local inflammation. Allam et al. demonstrated that anti-histone (and other anti-nucleosome) antibodies could block histone immune activity without any unexpected consequences to autoimmunity (34), and thus could be used as a novel biological agent for the treatment of autoimmune diseases such as shock, liver, and kidney failure, and even lupus. Therefore, understanding the details of histone’s dual role and targeting treatment can limit systemic damage and improve survival.

### Histones are promising antibacterial agents

The speed of discovery and development of antibiotics is stagnant. At the same time, the resistance of pathogens to antibiotics is increasing, resulting in a large number of fatal untreatable infections around the world. Therefore, there is an urgent need to develop new antibiotic strategies to combat this shocking reality. In addition to finding new antibiotics, strengthening research on existing natural host defense mechanisms may help researchers develop effective synthetic drugs that conform to biological principles. There is plenty of evidence that histones and histone fragments, as AMPs, directly eliminate bacteria, fungi, viruses, and parasites *in vitro* and *in vivo*, and their mechanism has begun to be precise. In general, extracellular histones play an antibacterial role like cationic AMPs. The primary mechanism by which histones exert the antibacterial effect *in vivo* is the primary component of neutrophil extracellular traps. Histones are expected to become a universal antibacterial, antiviral, and/or antifungal drug due to their highly conserved amino acid sequences among species, which provides a refreshing perspective to revitalize current antibiotic development strategies. It is worth noting that caution should be exercised when considering using histones as antimicrobials because histone itself is the cytotoxic and systematic injection of histone may be harmful to patients with life-aggravating infections (35, 136). However, studies have shown that during sodium chlorate treatment, the accumulation of circulating histones does not cause pathology, which challenges the idea that histones themselves are pathogenic (44). The application of histones as antimicrobial agents can be considered from two aspects. On the one hand, the level of free histones can be regulated by controlling the release of histones in NETs or lipid droplets to inhibit bacterial growth. On the other hand, safer and more effective histone and AMPs analogs can be developed to treat various infections.

### Prospects of histone modification therapy

Histone modification is a critical mechanism that regulates the expression of essential innate immune genes that sense responses to microbial pathogens. In the past decade, the role of histone acetylation and methylation in intrinsic immune cell function has been studied extensively. The reverse action of each enzyme reveals a complicated interplay of methyl and acetyl markers on a single promoter. However, it can be predicted that histone modification not only provides new perspectives and molecular biological tools for revealing the occurrence and development mechanisms of infection, inflammation, and autoimmune diseases, but also promotes the development of effective biotherapy for major clinical diseases. Therefore, it has a good application prospect.

## Author contributions

XL, YY, and YZ put forward the concept. XL and YZ studied literature and wrote manuscripts. XL and YY drew the figure and collected the tables. KP, ZZ, and LC contributed to language modification and content adjustment. XL, YY, and YZ participated in the revision of this manuscript. All authors read and approved the final manuscript.

## Funding

This study was supported by the National Natural Science Foundation of China (NO: 31370207, 81871256).

## Conflict of interest

The authors declare that the research was conducted in the absence of any commercial or financial relationships that could be construed as a potential conflict of interest.

## Publisher’s note

All claims expressed in this article are solely those of the authors and do not necessarily represent those of their affiliated organizations, or those of the publisher, the editors and the reviewers. Any product that may be evaluated in this article, or claim that may be made by its manufacturer, is not guaranteed or endorsed by the publisher.

## Abbreviations

DAMPs, damage-associated molecular patterns; PAMPs, pathogen-associated molecular patterns; PRRs, pattern recognition receptors; AMPs, antimicrobial peptides; NETs, neutrophil extracellular traps; DC, dendritic cell; Mg, Mycoplasma genitalium; HMTs, histone methyltransferases; HDMS, histone demethylases; HATS, histone acetyltransferases; HDACs, histone deacetylases; HDPs, host defense peptides; MUMP, murine microbial proteins; Pol-II, Polymerase II; MMP, matrix metalloproteinase; MTA, metastatic tumor antigen; IκBα, NF-κB inhibitor-α; ISG,IFN-stimulated gene; CRP: C-reactive protein; SARS-CoV-2, Severe Acute Respiratory Syndrome Coronavirus-2; H3Cit, Citrullinated histone 3; PAD, peptide arginine deiminase.
